# Percutaneous Transforaminal Endoscopic Lumbar Interbody Fusion: Clinical and Radiological Results of Mean 46-Month Follow-Up

**DOI:** 10.1155/2017/3731983

**Published:** 2017-02-27

**Authors:** Sang-Ho Lee, H. Yener Erken, Junseok Bae

**Affiliations:** ^1^Department of Neurological Surgery, Spine Health Wooridul Hospital, Seoul, Republic of Korea; ^2^Department of Orthopaedic Surgery, Spine Health Wooridul Hospital, Seoul, Republic of Korea

## Abstract

*Background*. Spinal fusion has been shown to be the preferred surgical option to reduce pain, recover function, and increase quality of life in the treatment of a variety of lumbar spinal disorders. The main goal of the present study is to report our clinical experience and results of percutaneous transforaminal endoscopic lumbar interbody fusion (PELIF) applications using the expandable spacer in a single institution.* Methods*. We performed a retrospective review of 18 patients with >12-month follow-up who had been operated on PELIF using expandable spacer from 2001 to 2007. Their clinical and radiological data were collected and analyzed.* Results*. The mean follow-up period was 46 months. The mean DH before the surgery was 8.3 mm which improved to 11.4 mm at the early postoperative period and regressed to 9.3 mm at the last follow-up visit. The VAS-B, VAS-L, and ODI scores at the last follow-up showed a 54%, 72%, and 69% improvement from the preoperative period, respectively.* Conclusions*. The presented PELIF technique with the expandable spacer seems to be a promising surgical technique for the treatment of a variety of lumbar spinal disorders. Conversely, radiological results including disc space subsidence make the stand-alone application of the expandable spacer debatable.

## 1. Introduction

Spinal fusion has been shown to be the preferred surgical option to reduce pain, recover function, and increase quality of life in the treatment of a variety of lumbar spinal disorders [1]. After Bagby [2] had first described lumbar interbody fusion (LIF) nearly 30 years ago, minimally invasive techniques for achieving LIF have become more popular over the past decade.

Recently, several studies [3–5] have shown that minimally invasive spinal (MIS) techniques, such as minimally invasive transforaminal interbody fusion (MI-TLIF), offer comparable results with the traditional open TLIF with the benefits of a shorter hospital stay, less blood loss, and shorter recovery time. This approach involves a paramedian incision and uses natural anatomical corridors by means of tubular retractor application, reducing the degree of muscle, fascia, and soft tissue injury compared to conventional open procedures. However, MI-TLIF still requires an open incision, partial laminotomy, facetectomy, and ligament flavum dissection in order to achieve a successful discectomy and effectively place the cage. The need for bone removal and open dissection may be eliminated when using the transforaminal posterolateral approach, which is the standard route for transforaminal endoscopic surgery. This approach, with a minimal access port through the Kambin triangle, provides nerve root protection using progressive soft tissue dilatators [6]. It uses a combination of endoscopic visualization and discectomy, expandable cage application, and long-acting local anesthetics with continuous sedation so that surgery can be performed without general anesthesia.

With the advancement of percutaneous LIF techniques a variety of medical equipment and cage designs, including the expandable B-Twin spacer, have been developed and the clinical use of these devices for various spinal disorders has been validated by multi- and single-center studies in the literature [7–14].

The main goal of the present study is to report our clinical experience and results of percutaneous endoscopic lumbar interbody fusion (PELIF) applications with the posterolateral transforaminal approach using the expandable spacer for a variety of spinal conditions in a single institution with a minimum of a 12-month postoperative follow-up period.

## 2. Material and Methods

After approval of the institutional review board (IRB), we performed a retrospective review of 18 patients who had been operated on with the described technique between 2001 and 2007. We included patients who were treated with the surgical technique described below into our study and who fulfilled the following inclusion criteria: patients suffering from a unilateral or a bilateral leg pain and/or lower back pain and who were diagnosed with a degenerative disc disease (DDD) with a collapsed disc, patients who were diagnosed with DDD with a primary or recurrent disc herniation, mild degenerative spondylolisthesis (SL) (grade 1), or failed back surgery syndrome (FBSS). We excluded patients who completed a postoperative follow-up period of less than 12 months. Patients who had additional central canal stenosis, diseases that impair bone quality (e.g., osteoporosis, other metabolic diseases, neoplasm, infection, or systemic diseases), a history of drug abuse, alcoholism, and/or noncompliance were also excluded from our study. Since evaluation of the outcomes would be debatable, the patients who were diagnosed with polyneuropathy (with or without the diagnoses mentioned above) using EMG-NCV studies were also excluded from this study.

The patients included in our study were evaluated for their baseline demographic characteristics, the pathologies for which they were being treated, surgical level, surgical time, length of hospital stay, and perioperative complications. We assessed clinical outcomes using the visual analogue scale (VAS) for back (VAS-B) and leg (VAS-L) pain at their preoperative examination, early postoperative clinical visits, and at the patients' final follow-up examination. We could also maintain patients' Oswestry disability index (ODI) scores at their preoperative and final follow-up examinations. Patient outcomes were graded as excellent, good, fair, and poor using a modified Macnab criteria [15] at the patients' final follow-up visits. Preoperative radiological studies included lumbar spine standing X-rays (standing anteroposterior and lateral neutral, flexion, and extension views), computerized tomography (CT), and magnetic resonance imaging (MRI) studies. Surgical times and duration of hospitalization were also recorded from patients' charts.

At the early postoperative and consequent follow-up examinations, we assessed radiological outcomes using lumbar spine standing X-rays (standing anteroposterior and lateral neutral, flexion, and extension views). Additional MRI or CT scans were only performed for patients with fair or poor clinical results or those with a VAS score > 4. On the standing lateral neutral X-rays, we measured the preoperative and postoperative disc height (DH) by measuring the distances between the inferior endplate of the cranial and superior endplate of the caudal vertebra at the middle and the posterior portion of the disc space and by calculating the average of these measurements. We also evaluated spinal instability on standing lateral neutral, flexion, and extension views. Measurement of slip was calculated using the method described by White III and Panjabi [16]. A slip of >3 mm in the neutral position, >3 mm translation, or >10 degrees angulation on flexion and extension views were defined as instability. Bone fusion was assessed with flexion-extension lateral radiographs. If there was no movement seen on the lateral view in flexion-extension in the index level and there was bony continuity of trabecular bridging, it was termed union. If there was any movement seen on the lateral view in flexion-extension or discontinuity of the trabecular bony bridging, it was termed nonunion. Radiological measurements were taken using digitalized tools in the PACS system, PiView STAR (Infinitt Co.Ltd., Seoul, Korea). Depending on these measurements, we defined patients as “Stable” and “Unstable.” Clinical evaluation at the final follow-up visit and all preoperative and postoperative radiological assessments were performed by an independent physician who was not involved in the surgical procedures.

Statistical analysis was performed using IBM SPSS (Statistical Package for Social Sciences) for Windows 22 software (IBM Corp, Armonk, NY). We used Students' *t*-tests to compare preoperative, early postoperative, and final follow-up VAS-B, VAS-L, and ODI scores, and DH. *p* value < 0.05 was considered to be statistically significant.

## 3. Surgical Technique

**Figure 1:**
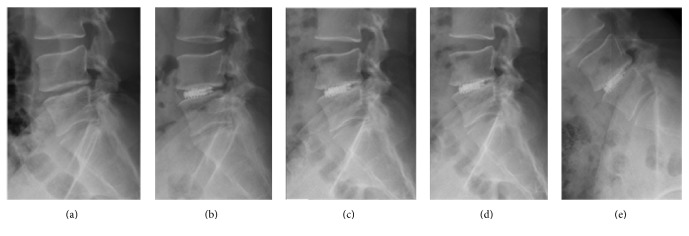
Representative case involving a patient with DDD and instability at the L4-5 level. (a-b) Fluoroscopic images showing disc removal using endoscopic forceps and endplate preparation using an endoscopic curette. (c) A trial implant without expansion in disc space is shown. (d-e) Fluoroscopic images showing the final construct with an expandable B-Twin spacer.

We performed the procedure in all of our patients using the posterolateral transforaminal approach. All of the procedures were performed in the prone position under local anesthesia with conscious sedation. Conscious sedation with midazolam and fentanyl allowed for continuous feedback from the patient during the entire procedure to avoid damage to the neural structures. Midazolam was administered in the dose of 0.05 mg/kg mg intramuscularly half an hour before surgery, followed by another dose intravenously during the operation, if required. Fentanyl dosage was 0.8 *μ*g/kg intravenously 10 minutes before the operation followed by additional doses intraoperatively, if required. An imaginary line drawn to the annular puncture site through the foramen designated the entry point into the skin and surgical trajectory. Prior to surgery, we used axial MRI and CT images to calculate the distance of the skin entry point of the needle from the midline and the needle trajectory. After infiltrating the intended needle entry tract with 8 mL to 10 mL of 1% lidocaine, an 18-gauge needle was inserted posterolaterally under fluoroscopic guidance. The location of nerve roots and the safe triangle were confirmed with the use of an epidurogram with radio opaque dye (Telebrix, Gluerbet, Aulnay-sous-Bois, France). After infiltrating 5 mL of 0.5% lidocaine on the surface of the annulus, the needle was advanced to the center of the disc space. Discography was performed by injecting 2-3 mL of a mixture of a radio opaque dye, indigo carmine (Carmine, United Korea Pharma., Yeongi, Korea), and normal saline mixed in 2 : 1 : 2 ratios. The needle was then replaced with a 0.8 mm guide wire. Progressive tissue dilatation was achieved through an 8 mm skin incision and a blunt cannulated obturator was passed over the guide wire under fluoroscopic control until its tip reached the outer surface of the annulus. We performed a reamed foraminoplasty in patients with foraminal stenosis and a collapsed disc space, in order to undercut the superior facet and to enlarge the foramen without touching or harming neural structures. We used bone reamers (Joimax GmbH, Karlsruhe, Germany) under the fluoroscopic guidance for foraminoplasty. A 7 mm beveled working cannula was placed over the obturator and advanced with twisting motions. After removing the degenerated nuclear material with a standard discectomy using pituitary forceps (Figure 1(a)), an end plate curettage was performed using curettes and rasps through the endoscopic cannula under endoscopic visualization for endplate preparation (Figure 1(b)). At this point in the surgery, our goal was to remove a minimum of 80% of the disc nucleus while maintaining the integrity of the annulus, so that it still contained the interbody implant. A trial implant without expansion was placed into the disc space and controlled by fluoroscopy to find the optimal location and size of the spacer (Figure 1(c)). After retrieving the trial implant, we placed demineralized bone matrix (DBM), cancellous bone allograft, or autogenous bone graft from the iliac crest through the endoscopic cannula into the anterior and lateral recesses of the disc space. Then, we introduced the B-Twin expandable spacer (B-Twin; Disc-O-Tech Medical Technologies Ltd., Herzliya, Israel) through the endoscopic cannula and expanded it under the lateral C-arm fluoroscopic control (Figures 1(d) and 1(e)). In the original description of this technique, it is recommended that surgeons not exceed the measured disc height by more than 10 ± 20% in order to avoid jeopardizing the annulus with excessive tension [12, 13]. We placed two expandable implants (one on the left side and one on the right) with a bilateral approach using the same technique in all the patients included in this study. The procedures at each side can be performed simultaneously or one after the other, depending on the surgeon's preference. We used endoscopic visualization to confirm the decompression of the exiting nerve root in all patients, as well as the decompression of the traversing nerve root in patients who had a disc herniation. Additionally, the exiting root could be mobilized under direct endoscopic vision with a flexible radiofrequency probe (Ellman; Ellman International LLC, USA). After retrieving the endoscope, the skin was sutured. We did not apply any additional percutaneous posterior fixation in any of our patients in this series.

The expandable spacer (B-Twin; Disc-O-Tech Medical Technologies Ltd., Herzliya, Israel) is made of titanium. When it is collapsed, the spacer is cylindrical in shape with a size of 5 mm in diameter and 25 mm in length. The expanded final shape is a trapezoid. There are three available sizes options: 9.5/11, 11.5/13, and 13.5/15. It is a trapezoid in cross section for the purpose of maintaining a physiological degree of lordosis in the involved segment. We selected the appropriate implant size according to the height of the collapsed disk, which was measured manually on the preoperative X-rays, CT, and MRI scans. The size was adjusted intraoperatively when necessary. The implant may be expanded up to 15 mm in diameter. After it is inserted into the disc space by a single-use delivery system, the device self-locks.

We generally permitted early ambulation in the upright position without forward flexion on the same day of surgery using a flexible lumbar orthosis. We discharged the patients on the same day or the day after surgery.

## 4. Results

The surgeries were performed on the L4-5 level in 13 patients, on the L5-S1 level in four patients, and on the L2-3 level in one patient. The mean age of the patients was 44.1 years (range, 26–63). The mean follow-up period was 46 months (range, 12–123). The average surgical time was 77 minutes (range, 62–100). The patients' preoperative diagnoses included degenerative disc disease (DDD) with disc herniation in nine patients (two recurrent, seven primary herniations), DDD in four patients, degenerative spondylolisthesis (SL) in two patients, FBSS in two patients, and DDD with instability in one patient, respectively.

The mean DH before the surgery was 8.3 ± 1.6 mm (range, 5.2–11.5) which improved to 11.4 ± 1.8 mm (range, 8.8–14.7) at the early postoperative period. The difference was significant (*p* < 0.05). Although the mean DH regressed to 9.3 ± 1.9 mm (range, 4.7–12.2) at the last follow-up visit, the difference between the last follow-up and the preoperative measurements still remained significant (*p* = 0.02). The regression of DH from the early postoperative to the last follow-up was statistically significant (*p* < 0.05). Additionally, we observed that the limbs of the implant were broken in five patients during the final follow-up X-ray controls.

The mean preoperative VAS-B and VAS-L pain scores were 6.5 ± 2.4 (range, 3.5–10) and 7.8 ± 2.0 (range, 4.7–10), respectively. The mean preoperative ODI was 69.9 ± 14.3 (range, 44.4–92). The mean VAS-B and leg VAS-L improved to 2.2 ± 0.9 (range 1–4.1) and 1.3 ± 0.8 (range 0–2.5), respectively, at the early postoperative period. The percentages of improvement at the early postoperative period from the perioperative period in VAS-B and VAS-L scores were 66% and 83%, respectively. The VAS-B, VAS-L, and ODI scores at the last follow-up examination were 3.0 ± 1.4 (range, 1–5.7), 2.2 ± 1.5 (range, 0–5), and 22.3 ± 17.1 (range, 4–71.1) with a 54%, 72%, and 69% improvement from the preoperative period, respectively. All differences between the preoperative and postoperative scores including the last follow-up examination scores were statistically significant (*p* < 0.05). The mean VAS-B and VAS-L scores at the final follow-up examination showed worse results than those during the early postoperative period, and the differences were statistically significant (*p* < 0.05). The demographic data and all results are summarized in Tables 1 and 2.

Fusion was achieved in 16 out of 18 patients who showed no segmental motion on their flexion-extension X-rays at their final follow-up examination. In one patient, nonunion and instability occurred accompanied by bone resorption at the superior endplate of the caudal vertebra. This patient had a two-level TLIF surgery above the surgical level three years before the current PELIF surgery. We recommended a revision surgery for this patient but she refused our advice because of her comorbidities and a high anesthetic risk. One patient experienced transitory dysesthesia but recovered fully three weeks after the surgery. The patient was treated with oral gabapentin in the dose of 75 mg three times a day for six weeks. Major complications such as a dural tear, CSF leakage, infection, and neurologic injury did not occur in any of the patients in our series. One patient needed a revision surgery with anterior lumbar interbody fusion (ALIF) and percutaneous pedicle screw fixation one month after the index surgery due to postoperative migration of one of the implants. At the final follow-up examination, seven patients rated their clinical results as excellent (41%), six patients as good (35%), three patients as fair (18%), and one patient as poor (6%) according to the modified Macnab criteria. The patient who was treated with an ALIF revision surgery one week after the index surgery was not included in the final follow-up clinical evaluations and statistical analyses.

All patients were ambulated on the same day of surgery. Mean postoperative time until hospital discharge was 25 hours (range, 12–50).

## 5. Discussion

It has previously been reported that MI-TLIF needs an incision of 30 mm and the time after surgery until ambulation and hospital discharge may be up to 3.2 days and 9.3 days on average, respectively [17]. In contrast, the PELIF approach reported in this study is performed through a skin incision of 7 mm length. All of our patients were ambulated on the same day after the surgery and were discharged from the hospital within 50 hours.

In the present study, we reported a unique technique and showed the practicality of PELIF. Although conventional fusion surgery is a gold standard for lumbar interbody fusion, we have performed PELIF only in limited patients who have spinal instability that should be corrected but are not able to undergo general anesthesia or prefer only for “local anesthetic” procedure. The use of endoscopy with progressive tissue dilatation allows for a less invasive approach than the classical MI-TLIF technique. It requires a small skin incision and avoids the removal of the facet joints, ligamentum flavum, and lamina which is required for a standard MI-TLIF. PELIF can be performed without general anesthesia. The use of conscious sedation decreases the side effects caused by general anesthesia and allows for patient-based neuromonitoring with continuous patient feedback. The distinctiveness of our technique is the endoscopy-based posterolateral approach which allows insertion of an interbody fusion cage through the foramen and Kambin's triangle without the need for any bone removal. A small foraminoplasty may be necessary particularly at the L5-S1 level, because of more difficult accessibility here than at the other levels due to the iliac crests and lumbar lordosis [4, 17].

Expandable cages allow indirect neural decompression by restoring intervertebral height. The B-Twin spacer that we used in our patients is a titanium expandable device with a lifting-lever mechanism which provides additional stability to the end plates on the axial plane, while preventing rotation and providing decent bone integration [12, 13]. Indeed, the biomechanical properties of the B-Twin implant provide mechanical restrictions in all planes, as its expansion creates distraction and stabilizes the intervertebral disc space by a lifting-lever mechanism. The initial penetration of the implant limbs to the end plates provides resistance to migration and axial rotation [8, 12, 13].

Previous reports using the B-Twin expandable spacer via a percutaneous approach have been published in the literature [8, 11–13]. In our study excellent or good results were obtained in 72.2% of the patients. Still, our satisfaction results are worse than the satisfaction results of other percutaneous LIF studies using the B-Twin expandable spacer in the literature (where 86%, 91%, and 83.2% of patients are reported as having excellent and good results) [8, 11, 12]. The small sample size of our patients might be the reason for this clinical difference as two of these previous studies had been performed as multicenter studies with 107 and 87 patients, respectively [8, 12].

Appropriate selection of patients and appropriate surgical indication of the procedure might be key factors in obtaining good results with the PELIF technique. In our series, we observed excellent or good results in three of the four patients who had previously been operated on with diagnoses of FBSS and DDD with recurrent HNP. Therefore, we may assume that the PELIF technique is more favorable for revision surgeries because it allows the surgeon to place the interbody cage in a previously-operated level while avoiding scar tissue and reducing the risk of neurological injury.

The collapsed diameter of the B-Twin spacer is 5 mm. Bearing this in mind, the PELIF technique may be challenging to perform through an extremely collapsed foramen with an intervertebral disc height < 5 mm, particularly in the L5-S1 level. Still, the described foraminoplasty in our report allows for the safe access to the disc space through the obstructing structures of the narrow foramen, such as hypertrophic facets, wide transverse processes, and osteophytes.

The postoperative recovery of our patients was fast and they were discharged from the hospital on an average of 25 hours after the surgery. The mean VAS-B, VAS-L, and ODI scores significantly improved at the early postoperative period and last follow-up examinations compared to their respective preoperative scores (*p* < 0.05). At the last follow-up examinations, the VAS-B, VAS-L, and ODI scores of our patients improved by 54%, 72%, and 69%, respectively, when compared to their respective preoperative examination scores. Although Folman et al. [12] reported slightly greater clinical improvement in VAS-B and VAS-L scores at the final follow-up (65% and 89%, respectively), our results are comparable to the reports of Xiao et al. [11] and Folman et al. [12], who also used the B-Twin expandable spacer via the percutaneous approach.

Our main concern after a stand-alone implantation of the B-Twin spacer might be the possibility of subsidence and disc space collapse because of the limbs of the B-Twin spacer penetrating the vertebral body endplates, or a possible migration of the implant. We observed one implant migration one month after the index surgery and performed a revision with a miniopen ALIF surgery with percutaneous pedicle screw fixation. Additionally, we observed nonunion and instability accompanied by bone resorption at the superior endplate of the caudal vertebra in one patient at the final follow-up examination 46 months after the index surgery. This patient was diagnosed with a mild degenerative SL and had a two-level interbody fusion above the current surgical level three years prior to this PELIF surgery. We believe it would have been a better option to augment the PELIF surgery with posterior pedicle screws or other posterior fixation methods in this patient.

**Figure 2:**
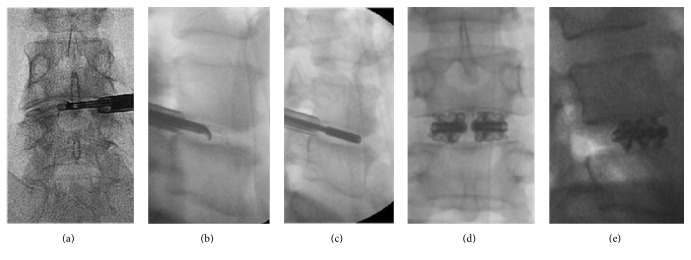
A 37-year-old male patient (patient number: 15 in the tables) with DDD and HNP at the L4-5 level. Lateral standing X-rays showing (a) preoperative, (b) early postoperative, and (c, d, e) final follow-up X-rays including standing lateral neutral, extension, and flexion views taken at 50 months after the surgery. Note that there is 1.9 mm reduction of the DH at the final follow-up examination compared to the early postoperative period. The operated level remained stable in extension and flexion views. The patient's VAS-B, VAS-L, and ODI scores were 1.4, 2, 4.4, respectively, at the final follow-up visit. The patient rated his result as “excellent.”

**Figure 3:**
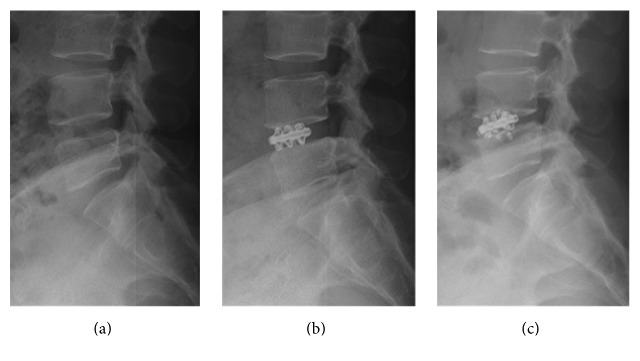
A 50-year-old female patient (patient number: 3 in the tables) with DDD at the L4-5 level. Lateral standing X-rays showing (a) preoperative, (b) early postoperative, and (c) final follow-up X-rays including standing lateral neutral views taken at 32 months after the surgery. It can be seen that the limbs of the implant are broken and there is a 2.5 mm reduction in DH at the final follow-up examination compared to the early postoperative period. The patient's VAS-B, VAS-L, and ODI scores were 4, 2.5, 31.1, respectively, at the final follow-up visit. The patient rated her result as “fair.”

The mean DH which was 8.4 mm before our procedure increased to 11.4 mm at the early postoperative period and decreased to 9.3 mm at the final follow-up examination. An average of 2.1 mm DH regression between patients' early postoperative period and their final follow-up examinations is remarkably greater than the 0.56 mm and 0.7 mm per level regression which have been reported after percutaneous B-Twin applications in the literature [11, 12]. However, this amount of subsidence did not jeopardize the stability of the implant in any of our patients (Figure 2). In all probability, the engagement of the limbs of the implant into the vertebral endplate provided a resistance against migration. The quality of the existing bone is most crucial in determining the ability to anchor the implant limbs into the vertebral endplate. This consideration may be of utmost importance when selecting candidates for PELIF. However, we observed subsidence even in younger patients. Therefore, subsidence cannot be attributed to poor bone quality alone. The special design of the implant with opening limbs which penetrates to the vertebral endplate providing a less implant-bone surface compared to the traditional interbody cages might be the main underlying cause of DH regression at the final follow-up examination. We also observed broken limbs of the implant in five patients at the final follow-up X-ray controls (Figure 3). Therefore, we recommend augmentation of the PELIF with percutaneous pedicle screw fixation or other minimally invasive posterior fixation methods particularly for patients who have degenerative SL, osteoporosis, or a previous adjacent-level fusion. One of the technical drawbacks of the B-Twin spacer placement is the irreversibility of the spacer expansion which makes relocation of the implant impossible when it has been incorrectly placed. In these circumstances, removal of the implant (particularly under continuous sedation) might prove to be very challenging. A new design for the expandable spacer, one that includes a removal mechanism, would be beneficial for these situations. Additionally, newer, expandable spacers with a greater bone-implant-bone surface contact than the current spacer have been reported in the literature [7, 9, 10, 14]. Further studies with longer follow-up periods using newer implants are necessary in order to make a definitive decision on the most appropriate implant design for PELIF.

Our study has some limitations that should be discussed. First, the sample size is too small and the mean postoperative follow-up period is not extensive enough to make definitive conclusions on clinical and radiological results. Prospective, multicenter studies comparing the percutaneous interbody fusion techniques to the traditional MIS techniques would allow for a better understanding of the rationale behind implementing this procedure. Eventually, this procedure should end up with a fusion rate that is comparable to other LIF techniques in order to be deemed successful. Definitive confirmation of fusion mass in more detail using 3-D CT scans would also be beneficial in proving the efficacy of our technique. In this study, patients were regularly evaluated using standing lumbar X-rays only. Additional MRI or CT scans were only performed on patients who represented fair or poor clinical results with a VAS score > 4. Therefore, we cannot make a definitive conclusion on the fusion rate of our patients. We can only conclude that all but one of our patients' surgical levels were stable on flexion-extension X-rays at the final follow-up examinations. Additionally, this procedure can be applied in a selected group patients and is not suitable for patients with severe central and lateral recess stenosis as endoscopic ventral decompression of soft disc can only achieve limited decompression. Osteoporosis needs to be carefully managed with further pedicle screws fixation to prevent subsidence and fracture of endplate.

## 6. Conclusions

The presented PELIF technique with the expandable spacer seems to be a promising surgical technique for treating patients suffering from DDD with or without disc herniation and instability, mild degenerative SL, and FBSS. Mean postoperative period until hospital discharge was faster than regular MI-TLIF techniques because of the less invasive nature of the PELIF approach. Clinical results of our small sample of patients are similar to previous reports using percutaneous LIF techniques. Conversely, radiological results including disc space subsidence in all and breakage of implant limbs in some of our patients make the stand-alone application of the expandable spacer (without any posterior fixation) debatable. We think that using newer expandable implant designs with a greater bone-implant-bone surface contact or applying PELIF with a minimally invasive posterior fixation method is worth considering for patients who need revision surgeries. We believe modifications in implant design are necessary improvements as the PELIF technique avoids scar tissue while ultimately reducing the risk of neurological injury in revision cases and eliminates the high risks of general anesthesia, especially in elderly patients.

## Figures and Tables

**Table 1:** Summary of the demographic data and results.

Number	Sex	Age yrs	Level	Last F/U months	Diagnosis	Radiological results last F/U complications and treatment	Satisfaction last F/U
1	M	28	L5-1	114	FBSS	Stable	Good
2	M	37	L5-1	101	DDD + recurrent HNP	Stable	Good
3	F	50	L4-5	32	DDD	Stable. Limbs of the implant were broken	Fair
4	F	53	L4-5	123	DDD + HNP	Stable. Limbs of the implant were broken	Good
5	M	52	L4-5	27	DDD + HNP	Stable	Excellent
6	M	33	L4-5	26	DDD + HNP	Stable. Limbs of the implant were broken	Excellent
7	F	62	L2-3	78	Degenerative SL	Stable	Good
8	M	47	L5-1	13	FBSS	Stable. Limbs of the implant were broken	Fair
9	F	47	L4-5	12	DDD + HNP	Stable	Fair
10	F	26	L4-5	24	DDD	Stable	Good
11	F	51	L4-5	23	DDD	Stable	Excellent
12	M	54	L4-5	16	DDD + recurrent HNP	Stable	Excellent
13	F	63	L4-5	46	Degenerative SL	Unstable, nonunion	Poor
14	F	26	L4-5	34	DDD + HNP	Stable. Limbs of the implant were broken	Good
15	M	37	L4-5	15	DDD + HNP	Stable	Excellent
16	M	41	L4-5	67	DDD + instability	Stable	Excellent
17	F	52	L4-5	32	DDD	Stable	Excellent
18	M	35	L5-1	—	DDD + HNP	Implant migration, revision with ALIF PPF	—

Note: F/U: follow-up, F: female, M: male, and yrs: years.

**Table 2:** Summary of the clinical and radiological results.

No	VAS-B			VAS-L			ODI		Disc height (mm)		
	Preop	Early PO	Last F/U	Preop	Early PO	Last F/U	Preop	Last F/U	Preop	Early PO	Last F/U
1	3.5	1.4	2	8.6	0	2	60	4.4	7.2	10.6	10.5
2	7.4	3.1	4	10	1.2	4	82	20	8.8	12.2	8.9
3	7.4	3.4	4	5.3	1.1	2.5	78	31.1	10.4	14.1	11.6
4	8.1	2.1	3	8.5	2.4	3.2	44.4	35.6	8.2	8.8	8.7
5	5.1	1.0	1.0	8.7	0	0	80	4	9.3	12.8	9.54
6	4.2	2.1	2.5	8.5	1	0	88	20	10.8	13.3	12.2
7	10	3.1	4.1	9.2	2.4	3.1	62	37.8	6.4	9.8	9.7
8	9.2	4.0	5.2	5.4	2.1	3.2	75	28	5.2	9.2	7.4
9	4.0	1.1	3.4	8.4	1.9	5.0	64.4	32	11.5	14.7	11.4
10	3.8	2.5	2.2	2.4	1.0	1.0	47	13.3	8.7	12.8	11.8
11	5.0	2.3	1.1	10	1.5	2.1	92	6.7	7.9	9.9	7.5
12	4.3	2.1	2.3	8.9	1.1	1.4	57.5	24	8.4	10.8	8.14
13	5.1	2.1	5.7	9.0	1.5	3.9	73.3	71.1	7.1	11.1	4.7
14	8.2	1.8	4.0	4.7	1.1	3.0	77.8	18	6.4	11.4	9.9
15	4	1.1	1.4	8.4	2.1	2.0	60	4.4	8.1	9.9	8
16	10	2.5	2.1	8.2	1.5	2.4	88	24	8.5	10.9	8.5
17	10	1.0	3.0	8	0	0	60	4	7.5	9.6	9.6
18	7.9	4.1	—	9	2.5	—	70	—	10.2	13.6	—

Mean	6.5 ± 2.4	2.2 ± 0.9	3.0 ± 1.4	7.8 ± 2.0	1.3 ± 0.8	2.2 ± 1.5	69.9 ± 14.3	22.3 ± 17.1	8.3 ± 1.6	11.4 ± 1.8	9.3 ± 1.9

Note: F/U: follow-up. PO: postoperative.
