# Corrigendum to “Percutaneous Transforaminal Endoscopic Lumbar Interbody Fusion: Clinical and Radiological Results of Mean 46-Month Follow-Up”

**DOI:** 10.1155/2017/3431257

**Published:** 2017-07-12

**Authors:** Sang-Ho Lee, H. Yener Erken, Junseok Bae

**Affiliations:** ^1^Department of Neurological Surgery, Spine Health Wooridul Hospital, Seoul, Republic of Korea; ^2^Department of Orthopaedic Surgery, Spine Health Wooridul Hospital, Seoul, Republic of Korea

In the article titled “Percutaneous Transforaminal Endoscopic Lumbar Interbody Fusion: Clinical and Radiological Results of Mean 46-Month Follow-Up” [1], the images of Figures 1 and 2 are reversed. They should be corrected as follows.

## Figures and Tables

**Figure 1:**
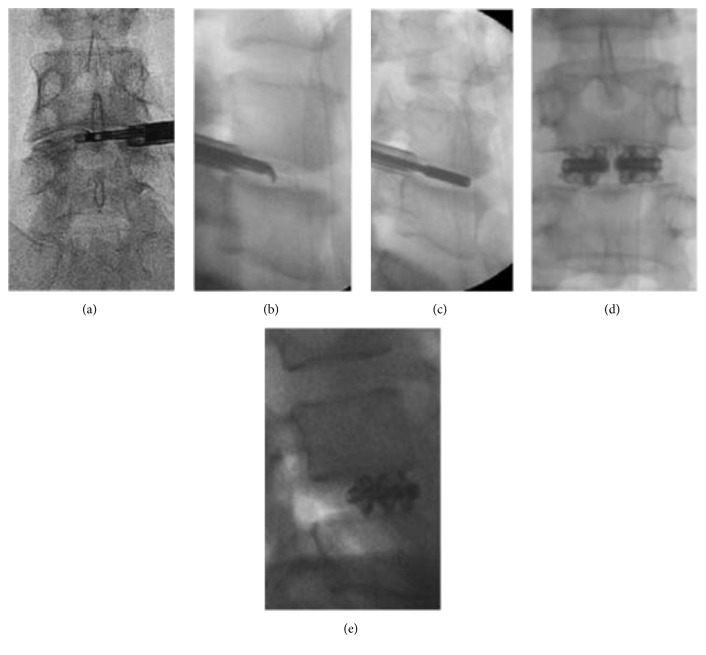
Representative case involving a patient with DDD and instability at the L4-5 level. (a-b) Fluoroscopic images showing disc removal using endoscopic forceps and endplate preparation using an endoscopic curette. (c) A trial implant without expansion in disc space is shown. (d-e) Fluoroscopic images showing the final construct with an expandable B-Twin spacer.

**Figure 2:**
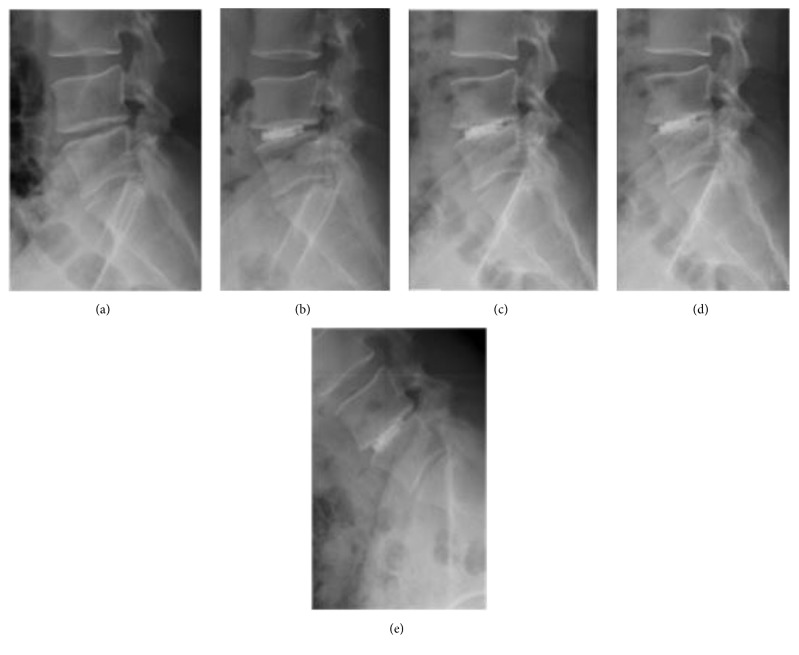
A 37-year-old male patient (patient number: 15 in the tables) with DDD and HNP at the L4-5 level. Lateral standing X-rays showing (a) preoperative, (b) early postoperative, and (c, d, e) final follow-up X-rays including standing lateral neutral, extension, and flexion views taken at 50 months after the surgery. Note that there is 1.9 mm reduction of the DH at the final follow-up examination compared to the early postoperative period. The operated level remained stable in extension and flexion views. The patient's VAS-B, VAS-L, and ODI scores were 1.4, 2, 4.4, respectively, at the final follow-up visit. The patient rated his result as “excellent.”
